# Barbell load distribution and lifting velocity affect bench press exercise volume and perceived exertion

**DOI:** 10.1371/journal.pone.0278909

**Published:** 2022-12-09

**Authors:** Carlo Ferri Marini, Vahid Shoaei, Lorenzo Micheli, Piergiorgio Francia, Tommaso Grossi, Serena Maggio, Piero Benelli, Ario Federici, Francesco Lucertini, Luca Zoffoli

**Affiliations:** 1 Department of Biomolecular Sciences, Division of Exercise and Health Sciences, University of Urbino Carlo Bo, Carlo Bo, Italy; 2 Scientific Research & Innovation Department, Technogym S.p.A., Italy; University of Belgrade: Univerzitet u Beogradu, SERBIA

## Abstract

**Objective:**

The intensity of barbell bench press exercise is generally prescribed as the load to be lifted for a specific number of repetitions; however, other factors (e.g., execution velocity) can affect bench press exercise intensity. Moreover, no study assessed whether load distribution (i.e., the distance between the disc stacks on the two sides of the barbell) affects exercise intensity. The present study aims to assess how different combinations of load, velocity, and barbell load distribution affect the number of repetitions to failure (REP_failure_), and rating of perceived exertion (RPE_fatigue_) and number of repetitions (REP_fatigue_) at fatigue onset.

**Methods:**

Ten males (age 23.3±1.8 years) performed bench press exercises to exhaustion using random combinations of three loads (50%, 65%, and 80% of 1 repetition maximum), three execution velocities (50%, 70%, and 90% of maximal concentric velocity), and two load distributions (narrow and wide). Three separate three-way repeated-measures ANOVAs were performed to assess the effect of load, velocity, and load distribution on REP_failure_, RPE_fatigue_, and REP_fatigue_ expressed as a percentage of REP_failure_.

**Results:**

REP_failure_ was affected by load (*p*<0.001), velocity (*p*<0.001), and distribution (*p* = 0.005). The interactions between load and velocity (*p*<0.001) and load and distribution (*p* = 0.004) showed a significant effect on REP_failure_, whereas the interaction between velocity and distribution was not significant (*p* = 0.360). Overall, more REP_failure_ were performed using lower loads, higher velocities, and a wider distribution. RPE_fatigue_ and REP_fatigue_ were affected by load (*p*<0.001 and *p* = 0.007, respectively) and velocity (*p*<0.001 and *p*<0.001, respectively), and not by distribution (*p* = 0.510 and *p* = 0.571, respectively) or the two-way interaction effects. Overall, using higher loads yielded higher RPE_fatigue_ but lower REP_fatigue_, while RPE_fatigue_ and REP_fatigue_ were higher when slower velocities were used.

**Conclusion:**

The current investigation shows that not only load but also velocity and barbell load distribution may influence bench press training volume and perceived exertion.

## Introduction

Resistance training is important for both health purposes and sports performance [1]. However, its effectiveness relies on the appropriate design of its constitutive parameters, such as weekly frequency, type of exercise, volume, and intensity [2]. In particular, exercise intensity plays a pivotal role in any resistance training protocol [3].

Resistance exercise intensity is usually prescribed as the load to be lifted, and it is traditionally expressed as a percentage of one-repetition maximum (1-RM), which is defined as the greatest resistance that can be lifted through the full range of motion in a controlled manner with good posture [2]. Despite 1-RM is still the standard for dynamic maximal strength assessment [2, 4], prescribing strength exercise using the same % of 1-RM can yield different intensities in different individuals. Indeed, there is high interindividual variability in the number of repetitions performed to failure at the same % of 1-RM [5, 6]. Consequently, other methods [7] have been proposed to prescribe resistance exercise intensity using a load that allows performing the desired number of repetitions at maximum before failure (multiple-RM) or one that produces a certain rate of perceived exertion (RPE).

Prescribing strength exercise intensity, however, remains a complex task. Indeed, studies [6, 8] have shown that resistance training parameters (i.e., number of repetitions to failure and RPE) are not only affected by the training load but also by the repetition velocity, which further increases the complexity of the resistance exercise intensity prescription.

Furthermore, the equipment used to exercise may also affect the prescription of exercise intensity. Indeed, studies reported that the use of different barbells can affect both exercise biomechanics and muscle activation [9, 10]. Moreover, no studies have investigated if varying the barbell length or the loading distributions (e.g., using different sets of discs on the same barbell) affects resistance training performance.

Another essential consideration when prescribing resistance exercise is whether the sets should be performed to muscular failure or not. In this regard, a recent systematic review on the topic showed that training to muscle failure does not seem to be required to increase muscle strength or size [11]. Indeed, international organizations (e.g., [12]) recommend, in certain instances, performing each set with the proper technique and to the point of muscle fatigue but not failure to reduce the chance of injury or debilitating muscle soreness [2, 13, 14]. In this regard, a recent systematic review on this topic [15] highlights that resistance training, when performed to failure, leads to a higher decline in biomechanical properties and metabolic responses. While determining muscle failure is a straightforward task (i.e., when a repetition cannot be completed with the proper technique), determining the point of muscle fatigue is a more complex task and several methods have been proposed over the years. These methods mostly rely on indicators such as the involuntary velocity loss during the set [16, 17] or psychological scales such as RPE [18, 19], which are associated with muscle fatigue and have also been proposed as methods for prescribing resistance exercise intensity. Although there are several studies assessing how resistance exercise parameters (e.g., load and velocity) affect exercise intensity (e.g., number of repetitions and RPE) at failure [6, 8], no study comprehensively assessed how these parameters affect exercise intensity at fatigue.

To date, there are no studies that evaluated how the loads, velocities, and load distributions affect resistance exercise intensity at failure and fatigue onset (i.e., involuntary velocity loss). Therefore, the aim of the present study is to assess if and how different combinations of loads, repetition velocities, and barbell load distributions affect both the number of repetitions to failure and the number of repetitions and RPE at fatigue onset during the bench press exercise.

## Materials and methods

### Participants

Ten physically active male students of the University of Urbino (Italy) were recruited for this study (age 23.3 ± 1.8 years; weight 79.5 ± 7.5 kg; height 1.82 ± 0.08 m; fat mass 13.1 ± 4.0%). The inclusion criteria were: a) a minimum of 2 resistance training sessions per week performed over the month before the test; b) at least 2 years of bench press exercise experience [2]; c) no history of upper limb injuries. As additional inclusion criteria, before the start of the study the participants underwent a medical examination, which also included a cardiopulmonary exercise test. All the participants obtained a medical clearance to perform maximal strength exercises. Subsequently, each participant provided written informed consent by signing the informed consent form indicating the project’s aims, testing procedures, and potential risks and discomfort associated with the testing procedures. The present study was approved by the institutional review board of the School of Sport Sciences of the University of Urbino (Italy) and was performed according to the Declaration of Helsinki.

### Equipment setup

All the tests carried out during the present study were performed on a flat bench (Technogym SPA, Cesena, Italy). Above and perpendicularly to the flat bench were placed an omnibus resistance exercise (OMNI-RES [20]) scale, and a linear encoder, connected to an analog/digital acquisition system (DAQ, ApLab, Rome, Italy) sampling at 2 kHz with 16-bit resolution. Both the OMNI-RES and the encoder were supported by adjustable support in order not to limit in any way the subjects’ freedom of movement and to ensure the positioning of the encoder perpendicular to the barbell while maintaining the OMNI-RES scale directly in front of the subjects during the exercise. Discs of various sizes (Technogym SPA, Cesena, Italy) were used (see Table 1) and a modified barbell was created to allow the disc holders to be moved and secured, using disc-locking springs, at any point of the barbell length.

**Table 1:** Physical characteristics of the barbell and discs used in the study.

	Mass (kg)	Length (mm)	Thickness (mm)
*Barbell*	12	2200	/
*20 kg disc*	20	/	38
*10 kg disc*	10	/	30
*5 kg disc*	5	/	24
*2*.*5 kg disc*	2.5	/	16
*1*.*25 kg disc*	1.25	/	16

### Study design

**Fig 1:**
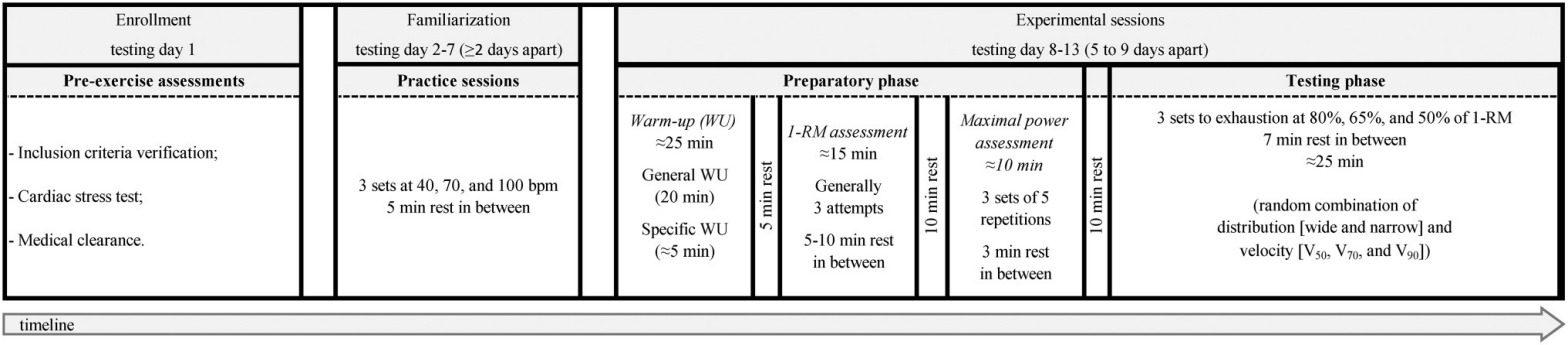
Experimental design and timeline of the pre-exercise assessment, familiarization and experimental sessions. bpm, beats per minute; 1-RM, 1 repetition maximum; 50% (V_50_), 70% (V_70_), and 90% (V_90_) of maximal concentric velocity.

In this cross-over study (see Fig 1 for a graphical representation of the design), six familiarization sessions followed by six experimental sessions were carried out to assess the effect of different combinations of resistance (*load*), execution velocity (*velocity*), and load distribution (*distribution*) on bench press training volume and RPE. For this purpose, during the experimental sessions, three load intensities corresponding to 50% (1-RM_50_), 65% (1-RM_65_), and 80% (1-RM_80_) of 1-RM were chosen to represent the intensity ranges typically used to train muscular endurance, hypertrophy, and strength [3]. For each load, three different velocities corresponding to 50% (V_50_), 70% (V_70_), and 90% (V_90_) of the maximal concentric velocity were used. Finally, for each *load* and *velocity* combination, a narrow (D_narrow_) or wide (D_wide_) disc *distribution* on the barbell (i.e., the separation between the two disc stacks) was used to represent, on average, the position of the disc stacks on barbells of different lengths available on the market.

The familiarization sessions were performed over three weeks with an interval between sessions of at least 48 hours. In general, all the familiarization sessions were not exhaustive, and focused on ensuring that the participants became accustomed to the proper exercise technique, the use of the acoustic pacer, and the reporting of the perceived effort during the exercise. On the other hand, each experimental session involved a significant number of sets, some of them performed to exhaustion. Therefore, to ensure that the results were not influenced by residual fatigue, the experimental sessions were carried out over a period of six weeks, five to nine days apart, and under controlled room temperature and humidity at the same time of the day. For the whole duration of the study, participants were asked not to change their diet and not to take any type of drugs or supplements that could have affected their performance capacity or their perception of effort.

### Familiarization sessions

During the familiarization sessions, the participants were trained on the correct execution of the exercise and on the utilization of the OMNI-RES scale. Particular attention was paid to ensuring that all participants performed the "flat bench press" exercise following the NSCA guidelines [21]. In particular, great attention was paid to ensure that: a) the shoulder blades were adducted and touched the bench along with the pelvis; b) the feet were firmly and well-parted on the ground; c) a pronated grip was used with the hands at 150% of the acromion-acromion distance [22]; d) the barbell was lifted from the chest until the elbows were fully extended (concentric phase); e) the barbell went down in a controlled movement until it touched the chest near the nipples (eccentric phase).

In addition, participants were instructed to mentally evaluate the level of effort during the concentric phase of each repetition and then verbally provide it during the eccentric phase [23]. Specifically, they were instructed not to perform isometric phases between the concentric and eccentric phases and were familiarized with following the cadence of a metronome (two beats per movement phase), which was used to control execution velocity. During familiarization sessions, the execution times were arbitrarily set at 40, 70, and 100 bpm for the first, second, and third sets respectively, which were performed with 5 minutes of rest in between sets. Great care was dedicated to ensuring that the participants were well accustomed with the use of an acoustic pacer and in providing reliable feedback about their perceived effort during the exercise without affecting the execution of the repetitions.

### Experimental sessions

**Fig 2:**
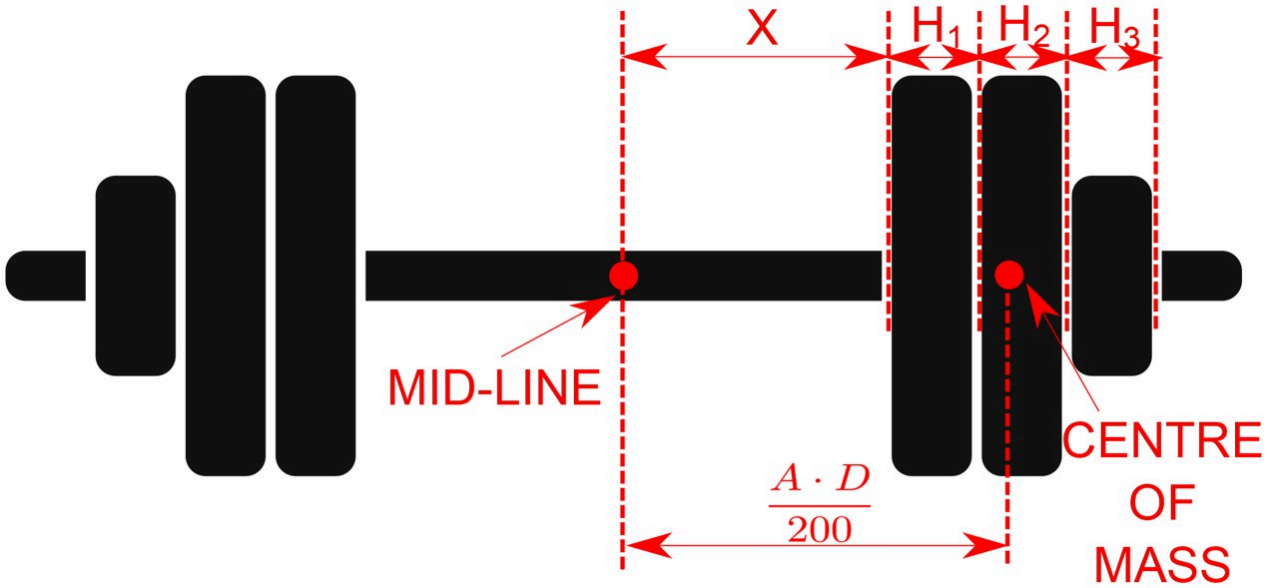
Graphical representation of discs’ distribution on the barbell. X, distance between mid-line and the disc; H1, thickness of the first disc; H2, thickness of the second disc; H3, thickness of the third disc; A, the acromion-acromion distance; D, distribution of the disc stacks on the two sides of the barbell corresponding to a distance equal to either 275% or 325% of A.

During each visit, the participants followed a standard protocol consisting of a preparatory phase and a testing phase. For each experimental session, one *distribution* (D_narrow_ or D_wide_) was randomly chosen and used for the whole session. Load distribution represents the distance between the centre of masses of the two disc stacks on the sides of the barbell, which corresponds to 275% (D_narrow_) or 325% (D_wide_) of the acromion-acromion width. These distances were selected according to preliminary pilot testing, where the in-between distance of the discs pack of both olympic (2.2 m) and a short (1.5 m) barbell-length were percentualized to the acromion-acromion width of the participants. For each training load, the exact positioning of the discs was obtained by: a) calculating the minimum number of discs required to achieve the target load; b) loading the discs in descending order by weight [24]; c) ensuring that the discs were equally divided between the two sides of the barbell. These constraints allowed the calculation of the distance X from the barbell mid-line where to place the discs in order to generate an overall moment arm equal to the desired percentage of the acromion-acromion distance. The mathematical derivation of this procedure led to the application of the following equation (see Fig 2 for a graphical representation):

$$X\;[mm]=\frac{A\;\left[mm\right]\cdot D_{narrow/wide}\;[\%]}{200}-\frac{\sum_{i=1}^{n}\left(\frac{H_{i}\;[mm]}{2}+\sum_{j=1}^{i-1}H_{j}\;[mm]\right)\cdot W_{i}\;[kg]}{\sum_{i=1}^{n}W_{i}\;[kg]}$$

where *A* is the acromion-acromion distance; *n* is the number of discs to be added to each side of the barbell; *H*_*i*_ is the thickness of the i^th^ disc; *W*_*i*_ is the mass of the i^th^ disc.

#### Preparatory phase

The preparatory phase consisted of a general and a specific warm-up, followed by the 1-RM and maximal power assessments.

*Warm-up*. The general warm-up [25] was performed on the cycle ergometer by cycling for 20 minutes at an exercise intensity corresponding to 60% of the estimated maximal heart rate [26]. The specific warm-up consisted of a set of 20 repetitions on a flat bench with the barbell unloaded, followed by a set of 8 repetitions with a load equal to about 50% of the estimated 1-RM and a set of 4 repetitions with a load equal to about 70% of the estimated 1-RM [25].

*1-RM assessment*. After about 5 minutes of rest [25], participants’ 1-RM was assessed [21]. Briefly, participants attempted to lift the load equal to the estimated 1-RM and, if one repetition was completed, the load was increased, whereas if the participant could not perform a repetition the load was decreased. In both cases a new attempt was made and the procedure was repeated until 1-RM was identified. Generally, 1-RM was identified within 3 attempts. Ferreira et al. [27] suggested using 7 minutes of rest to maximize performance during repeated bench press exercises. Accordingly, that time was adopted as reference, although occasional adjustments within the 5 to 10 minutes range were allowed to cope with participants’ feedback about their readiness for the next 1-RM attempt.

*Maximal power assessment*. After 10 minutes of rest, maximal power was assessed. Participants were asked to perform 3 sets (3-min apart) of 3 repetitions each, with the *load* (calculated as the corresponding percentage of the 1-RM load obtained during the 1-RM assessment) of 1-RM_80_, 1-RM_65_, and 1-RM_50_, respectively. During the 3 sets, participants were asked to try to achieve the maximal concentric velocity in each repetition. For each set, the average concentric velocity of the fastest repetition was then used for the calculation of the three V_50_, V_70_, and V_90_ lifting *velocities* to be used, for each *load*, during the testing phase. Finally, for each *load*, the measurements obtained by the linear encoder during the maximal power assessment test were used to identify the three repetition cadences to be set on the metronome in order to obtain the three *velocities*. A dedicated algorithm was designed to: a) smooth the position readings of the linear encoder by a 4^th^ order low-pass Butterworth filter with phase correction and cut-off frequency set at 6 Hz; b) detect the relative maximums and minimums in the smoothed signal to obtain respectively the beginning and the end of each concentric phase; c) calculate the time elapsed between each maximum and minimum; d) obtain the minimum elapsed time and convert it to bpm considering 2 beats for the completion of the concentric phase; e) multiply the resulting value by the three desired *velocities*. The whole procedure was coded within a MATLAB script (ver. 2014a, MathWorks, Inc., Massachusetts, USA), which was used to properly set up the subsequent testing phase.

#### Testing phase

Ten minutes after the end of the preparatory phase, the participants performed three sets to exhaustion with 1-RM_80_, 1-RM_65_, and 1-RM_50_ *loads*. Each of these sets was carried out with a specific combination of *velocity* and *distribution*, which was randomly selected for each experimental session as one of the six possible combinations of *velocity* (V_50_, V_70_, and V_90_) and *distribution* (D_narrow_ and D_wide_).

During each set, participants were asked to verbally provide the RPE at each repetition starting from the moment in which they were no longer able to sustain the repetition cadence due to fatigue onset. The compliance with the repetition cadence, hence the repetition corresponding to fatigue onset, was assessed visually and audibly by the operators. For each set performed, the number of repetitions to failure (REP_failure_), the RPE at fatigue onset (RPE_fatigue_), and the number of repetitions at fatigue onset, which was expressed as a percentage of REP_failure_ (%REP_fatigue_), were recorded and used in the following analyses.

Two operators assisted the participants at the two extremities of the barbell, while a third one noted the RPE values provided by the subject. The sets were separated by about 7 minutes of rest [27].

### Statistical analysis

A three-way repeated-measure ANOVA was performed to assess if the REP_failure_ (dependent variable) were affected by *load* (1RM_50_, 1RM_65,_ and 1RM_80_), *velocity* (V_50_, V_70_, and V_90_), and *distribution* (D_narrow_ and D_wide_). Two separate three-way repeated-measures ANOVAs were also performed to assess the effect of *load*, *velocity*, and *distribution* (independent variables) on RPE_fatigue_ and %REP_fatigue_ (dependent variables).

The assumption of sphericity was assessed in each ANOVA using Mauchly’s sphericity test, and when the assumption was not met, the Greenhouse-Geisser correction was used. When significant effects were found, the Bonferroni corrected post-hoc pairwise comparisons were performed. In addition, for each test the corresponding partial eta-square (*η*_*p*_^2^) was calculated as a measure of effect-size.

Statistical analyses were performed using the SPSS Statistics (IBM, v.20) software, with an *α* level of statistical significance of 0.05.

## Results

REP_failure_, RPE_fatigue_, and %REP_fatigue_ mean values, along with their SD and CV, are presented in Table 2 (raw individual data can be found in S1 File).

**Table 2:** REP_failure_, RPE_fatigue_, and %REP_fatigue_ descriptive statistics during bench press exercises performed with different load distributions, velocities, and loads.

	REP_failure_			RPE_fatigue_			%REP_fatigue_		
	Mean	SD	CV	Mean	SD	CV	Mean	SD	CV
Distribution									
*D*_*Narrow*_	12.2	6.8	0.56	8.2	0.9	0.11	81.0	12.5	0.15
*D*_*Wide*_	13.2	7.5	0.57	8.1	0.9	0.11	81.8	12.4	0.15
Velocity									
V_50_	11.0	5.9	0.53	8.6	0.9	0.11	88.2	10.0	0.11
V_70_	12.7	7.2	0.57	8.2	0.8	0.10	82.4	11.4	0.14
V_90_	14.2	8.0	0.56	7.7	0.7	0.09	73.5	11.2	0.15
Load									
1-RM_50_	21.2	3.5	0.16	7.9	0.8	0.10	86.1	7.2	0.08
1-RM_65_	12.1	1.8	0.15	8.2	0.8	0.10	82.0	10.2	0.12
1-RM_80_	4.8	1.3	0.28	8.4	0.9	0.11	76.1	16.1	0.21

REP_failure_, number of repetitions performed to failure; RPE_fatigue_, rate of perceived exertion at fatigue onset (i.e., involuntary reduction of lifting velocity); %REP_fatigue_, number of repetitions at fatigue onset expressed as a percentage of REP_failure_; SD, standard deviation; CV, coefficient of variation; Load, load corresponding to 50% (1-RM_50_), 65% (1-RM_65_), and 80% (1-RM_80_) of 1 repetition maximum; Velocity, velocities corresponding to 50% (V_50_), 70% (V_70_), and 90% (V_90_) of the maximum concentric velocity; Distribution, narrow (D_narrow_) and wide (D_wide_) load distribution on the barbell.

### REP_failure_

REP_failure_ was affected by *load* (F_(1.243, 11.185)_ = 735.096, *p* < 0.001, *η*_*p*_^2^ = 0.988), *velocity* (F_(2, 18)_ = 51.098, *p* < 0.001, *η*_*p*_^2^ = 0.850), and *distribution* (F_(1, 9)_ = 13.450, *p* = 0.005, *η*_*p*_^2^ = 0.599).

The interactions between *load* x *velocity* (F_(4, 36)_ = 29.760, *p* < 0.001, *η*_*p*_^2^ = 0.768) and *load* x *distribution* (F_(2, 18)_ = 7.416, *p* = 0.004, *η*_*p*_^2^ = 0.452) showed a significant effect on REP_failure_, whereas the interaction between *velocity* x *distribution* was not significant (F_(2, 18)_ = 1.083, *p* = 0.360, *η*_*p*_^2^ = 0.107).

As shown in Table 3, the post-hoc pairwise comparisons of the main effects showed that participants performed more REP_failure_ when the bench press exercises were performed using lower *loads*, higher *velocities*, and wider *distribution*.

**Table 3:** Post-hoc pairwise comparisons of the main effects between REP_failure_, RPE_fatigue_, and %REP_fatigue_ at different load distributions, velocities, and loads.

	REP_failure_				RPE_fatigue_				%REP_fatigue_			
	MD	CI_INF_	CI_SUP_	*p*	MD	CI_INF_	CI_SUP_	*p*	MD	CI_INF_	CI_SUP_	*p*
Distribution												
*D*_*narrow*_−*D*_*wide*_	-1.0	-1.6	-0.4	0.005	0.1	-0.2	0.5	0.510	-0.8	-3.5	1.9	0.517
Velocity												
V_50_ –V_70_	-1.7	-2.4	-1.0	<0.001	0.3	0.1	0.6	0.019	5.8	-0.7	12.2	0.081
V_50_ –V_90_	-3.2	-4.2	-2.2	<0.001	0.8	0.4	1.2	<0.001	14.7	8.8	20.6	<0.001
V_70_ –V_90_	-1.5	-2.6	-0.4	0.009	0.5	0.1	0.9	0.011	8.9	5.6	12.2	<0.001
Load												
1-RM_50_−1-RM_65_	9.2	8.1	10.2	<0.001	-0.2	-0.5	0.0	0.100	4.1	0.6	7.6	0.022
1-RM_50_−1-RM_80_	16.5	14.8	18.1	<0.001	-0.5	-0.8	-0.2	0.003	10.0	2.5	17.4	0.011
1-RM_65_−1-RM_80_	7.3	6.4	8.2	<0.001	-0.3	-0.6	0.0	0.077	5.9	-2.2	14.0	0.187

REP_failure_, number of repetitions performed to failure; RPE_fatigue_, rate of perceived exertion at fatigue onset (i.e., involuntary reduction of lifting velocity); %REP_fatigue_, number of repetitions at fatigue onset expressed as a percentage of REP_failure_; MD, mean difference; inferior (CI_INF_) and superior (CI_SUP_) 95% confidence interval; Load, load corresponding to 50% (1-RM_50_), 65% (1-RM_65_), and 80% (1-RM_80_) of 1 repetition maximum; Velocity, velocities corresponding to 50% (V_50_), 70% (V_70_), and 90% (V_90_) of the maximum concentric velocity; Distribution, narrow (*D*_*narrow*_) and wide (*D*_*wide*_) load distribution on the barbell.

For each dependent variable REP_failure_, RPE_fatigue_, and %REP_fatigue_, the pairwise comparisons are based on the estimated marginal means derived from separate three-way (distribution, velocity, and load) repeated-measures ANOVAs and *p* and CI values were adjusted for multiple comparisons using Bonferroni.

The post-hoc pairwise comparisons of the interaction between *load* x *velocity* showed that REP_failure_ performed at the three velocities differed from each other at 1RM_50_ and 1RM_65_, whereas at 1RM_80_ only the fastest and slowest velocities were different.

When the post-hoc pairwise comparisons of the interaction between *load* x *distribution* were considered, REP_failure_ were, on average, higher when the wider *distribution* was used with the lighter *loads*, with significant REP_failure_ differences between wide and narrow *distribution* at 1RM_50_ (1.6±2.6, *p* = 0.002) and 1RM_65_ (1.1±2.0, *p* = 0.026), but not at 1RM_80_ (0.3±1.4, *p* = 0.159).

### RPE_fatigue_ and %REP_fatigue_

RPE_fatigue_ and %REP_fatigue_ were affected by *load* (RPE_fatigue_: F_(2, 18)_ = 12.656, *p* < 0.001, *η*_*p*_^2^ = 0.584; %REP_fatigue_: F_(1.293, 11.635)_ = 9.690, *p* = 0.007, *η*_*p*_^2^ = 0.518) and *velocity* (RPE_fatigue_: F_(2, 18)_ = 24.427, *p* < 0.001, *η*_*p*_^2^ = 0.731; %REP_fatigue_: F_(2, 18)_ = 32.327, *p* < 0.001, *η*_*p*_^2^ = 0.782), but not by *distribution* (RPE_fatigue_: F_(1, 9)_ = 0.472, *p* = 0.510, *η*_*p*_^2^ = 0.050; %REP_fatigue_: F_(1, 9)_ = 0.454, *p* = 0.571, *η*_*p*_^2^ = 0.048). The two-way interaction effects were not statistically significant in either RPE_fatigue_ or %REP_fatigue_.

RPE_fatigue_ and %REP_fatigue_ were higher when the sets were performed using slower *velocities* (Table 3), while higher bench press *loads* showed higher RPE_fatigue_ but lower %REP_fatigue._

## Discussion

The main finding of the present investigation was that *load*, *velocity*, and *distribution* affect REP_failure_. The results of the present study are consistent with the literature available on this topic [6, 8, 28], and show that more REP_failure_ can be performed at higher velocities and lower loads. In particular, Hatfield et al. [6] showed that using a very slow execution velocity (10-second concentric and 10-second eccentric phase per repetition) compared to a self-selected execution velocity leads to a significant difference (*p* ˂ 0.05) in the number of repetitions performed using the same load, expressed as a percentage of 1-RM. In this regard, a study from Sakamoto and Sinclair [8] confirmed that the number of repetitions performed is affected by movement velocity. Specifically, when four different execution velocities (slow, medium, fast, and ballistic) were used, there was a significant interaction between velocity and load (*p* ˂ 0.001), with higher number of repetitions performed with faster velocities and lower loads, whereas the effect of velocity at higher loads was relatively smaller [8]. These results are in line with those of the present study, showing a significant interaction between load and velocity (*p* ˂ 0.001). Therefore, this implies that the training volume, expressed as load multiplied by the number of repetitions, is significantly reduced when the velocity is lower. Moreover, this is the first study to show that REP_failure_ is also affected by the load *distribution*, which in turn has significant interactions with the load. Specifically, the present study revealed that, on average, D_wide_ (i.e., with the weights further away from the hands) allowed the subjects to perform more repetitions (1.6, 1.1, and 0.3 more repetitions at 1RM_50_, 1RM_65_, and 1RM_80_, respectively) compared to D_narrow_, especially with lighter loads. Compared to D_narrow_, D_wide_ increases the distance between the disc stacks and the pivot point, which results in a longer moment arm of the discs at each side of the barbell [29]. This might ease the subject in maintaining the barbell orientation, as greater torque would be required to alter the barbell orientation. It is well known that performing resistance exercises on unstable surfaces reduces the ability to generate force and increases the activity of the trunk and movement stabilizing muscles at the expense of the prime movers [30]. Likewise, the D_wide_ distribution might have facilitated the barbell stability during the exercise, which likely improved the overall movement efficiency, hence resulting in the higher number of performed repetitions that emerged from the current study. These results highlight that using solely percentages of 1-RM (i.e., *load*) to prescribe a specific number of repetitions, without accounting for *velocity* and *distribution*, might result in unexpected exercise intensities and training volumes.

Previous studies [6, 31] have already highlighted the interdependent association of repetition velocity, perceived exertion, training load and volume. However, to the best of our knowledge, the present study is the first to investigate how *load*, *velocity*, and *distribution* affect RPE_fatigue_ and %REP_fatigue_, showing that, while REP_failure_ is affected by both *load*, *velocity*, and *distribution*, RPE_fatigue_ and %REP_fatigue_ are affected by *load* and *velocity* but they are not altered by *distribution*. This suggests that an appreciable effect of *distribution* occurs only close to muscle failure, which adds further support to its role in stabilizing the barbell during the bench press exercise. Indeed, since higher or lower stability demands impact the movement stabilizers [32], the use of a more stable load distribution would likely affect the exercise performance especially when the fatigue impairs the ability to control the barbell. At fatigue onset, as *load* increases, RPE_fatigue_ increases while %REP_fatigue_ decreases, whereas as *velocity* decreases RPE_fatigue_ and %REP_fatigue_ increase. The effect of training load, which induces opposite RPE_fatigue_ and %REP_fatigue_ trends, reveals the importance of considering training loads as a possible confounding variable when the association between RPE and other indicators (e.g., the predicted number of repetitions that can be performed before muscular failure [19]) are used to prescribe exercise intensity [33]. In this regard, Zourdos et al. investigated the relation between perceived exertion and execution velocity, and observed that average velocity at all % of 1-RM had a strong inverse correlation with RPE in both expert resistance training subjects (*r =* -0.88, *p* ˂ 0.001) and novice resistance training athletes (*r =* -0.77, *p* = 0.001). These results are in line with the results of the present study and suggest that associations between load, velocity, and fatigue (in terms of perceived exertion) exist. Indeed, predicting the number of repetitions that can be performed before muscular failure, by estimating the repetitions in reserve (RIR), has gained recent interest in resistance training and it was recently proposed by National Strength and Conditioning Association [34] as an alternative method for prescribing resistance training load in nonfailure resistance training. However, the present study further adds to the complexity of the association between perceived exertion and RIR. Indeed, RIR estimation might not only be affected by the individuals’ experience in using RIR (e.g., the accuracy of RIR prediction improves with practice [35]), but also by other resistance training parameters, such as training load. Therefore, considering the beneficial effect of nonfailure resistance training (e.g., muscle hypertrophy and strength [36]) a more accurate understanding of the associations among RIR, perceived exertion, and resistance training parameters is warranted.

The results of the present study emphasize once more the importance of taking into account not only the training load, but also other exercise parameters such as velocity [37], as determinant factors of the training volume and RPE.

Although velocity loss can be considered an important marker of fatigue [16], a limitation of the present study is the arbitrary choice of using it uniquely as the marker of fatigue onset, which was assessed by the researchers as the incapacity of the subject to sustain the repetition cadence imposed by the metronome. Furthermore, the failure to maintain the desired movement pace has been previously adopted as a criterion for fatigue onset (e.g., [38]). Importantly, it was observed that velocity loss was greater in upper-limb exercises compared with lower-limb exercises [15]. Therefore, the results of the present study should be cautiously extrapolated for different muscle groups or movement patterns.

In the present study, during the testing phase, the order of the loads used for the three sets was not randomized. However, a long resting time between sets was used and the order was chosen after an extensive pilot testing phase. Specifically, pilot tests revealed that the intra-set decreasing load strategy yielded lower fatigue-related impairments in the subsequent sets and thus was chosen for the study. Therefore, this only partial randomization of load, velocity, and distribution was preferred to full randomization because it allowed us to limit the carryover effect of the previously performed sets and to reduce the testing burden (i.e., avoiding performing solely one set per testing session).

On the contrary, a strength of the present study is that 1-RM and maximal concentric velocity of each *load* were assessed in each testing session and used to select the experimental loads and velocities. Indeed, due to day-to-day fluctuations in resistance training performance (which can be caused by several factors, such as training-related fatigue, food and fluid intake, sleep quantity and quality, and psychological stress), using a predetermined 1-RM score could have led to a misrepresentation of the performance capacity of a subject on a given day [39].

The present study did not assess the underlying mechanisms (e.g., agonists, antagonists, and stabilizing muscle activation) or the effect of certain individual characteristics (e.g., age or muscle composition), which can affect muscle fatigue [40]. Therefore, future studies assessing how these factors are affected by *distribution*, *load*, and *velocity* are warranted. Additional studies are also needed to assess the importance of *load*, *velocity*, and *distribution* in both testing (e.g., 1- and multiple- RM test) and training (both at muscle fatigue and failure) settings.

## Conclusion

The present study shows that considering only the training load as a predictor of the exercise training volume might be misleading. Particularly for the bench press, not only the load, but also the way it is distributed on the barbell and the lifting velocity can influence the total training volume and perceived exertion. Using higher velocities and wider load distributions on the barbell allowed the participants to perform more repetitions to muscle failure, probably because of the elevated inertial forces required to de-stabilize the barbell under these conditions, which in turn facilitated the achievement of a higher number of repetitions. Additionally, the present study confirms the importance of *load* and *velocity* not only for prescribing resistance exercise at muscle failure but also at fatigue. Indeed, different combinations of these acute training variables might affect the volume and perceived exertion of resistance training programs, which could have implications on training adaptations and adherence.

## Supporting information

S1 File Participants’ REP_failure_, RPE_fatigue_, and %REP_fatigue_ during bench press exercises performed with different load distributions, velocities, and loads. REP_failure_, number of repetitions performed to failure; RPE_fatigue_, rate of perceived exertion at fatigue onset (i.e., involuntary reduction of lifting velocity); %REP_fatigue_, number of repetitions at fatigue onset normalized as a percentage of REP_failure_; SD, standard deviation; Load, load corresponding to 50% (1-RM_50_), 65% (1-RM_65_), and 80% (1-RM_80_) of 1 repetition maximum; Velocity, velocities corresponding to 50% (V_50_), 70% (V_70_), and 90% (V_90_) of the maximum concentric velocity; Distribution, narrow (D_narrow_) and wide (D_wide_) load distribution on the barbell.
(XLSX)
